# Supplementary material for: Ferritin triggers neutrophil extracellular trap-mediated cytokine storm through Msr1 contributing to adult-onset Still’s disease pathogenesis
Source: Nat Commun. 2022 Nov 10;13:6804. doi: 10.1038/s41467-022-34560-7 (PMC9648446; doi:10.1038/s41467-022-34560-7)
Supplement: Supplementary file 1 — Supplementary Information [file 41467_2022_34560_MOESM1_ESM.pdf]

Ferritin triggers neutrophil extracellular trap-mediated cytokine storm through Msr1 contributing to adult-onset Still's disease pathogenesis

Jinchao Jia[1,†], Mengyan Wang[1,†], Jianfen Meng[1,†], Yuning Ma[1, †], Yang Wang[2], Naijun Miao[2], Jialin Teng[1], Dehao Zhu[1], Hui Shi[1], Yue Sun[1], Honglei Liu[1], Xiaobing Cheng[1], Yutong Su[1], Junna Ye[1], Huihui Chi[1], Tingting Liu[1], Zhuochao Zhou[1], Liyan Wan[1], Xia Chen[1], Fan Wang[1], Hao Zhang[1], Jingjing Ben[3,*], Jing Wang[2,*], Chengde Yang[1,*], Qiongyi Hu[1,*]

[1]Department of Rheumatology and Immunology, Ruijin Hospital, Shanghai Jiao Tong University School of Medicine, Shanghai, China.

[2]Shanghai Institute of Immunology, Department of Immunology and Microbiology, Shanghai Jiao Tong University School of Medicine, Shanghai, China.

[3]Department of Pathophysiology, Key Laboratory of Cardiovascular Disease and Molecular Intervention, Nanjing Medical University, Nanjing, China.

Jinchao Jia, Mengyan Wang, Jianfen Meng, and Yuning Ma contributed equally to this work.

Corresponding author: Qiongyi Hu or Chengde Yang or Jing Wang or Jingjing Ben

Qiongyi Hu, MD, PhD

Department of Rheumatology and Immunology, Ruijin Hospital, Shanghai Jiao Tong University School of Medicine, No. 197 Ruijin Second Road, Shanghai 200025, China

Tel.: (86)-21-64370045ext665130; Fax: (86)-21-54109718; Email: huqiongyi131@163.com

Chengde Yang, Email: yangchengde@sina.com; Jing Wang, Email: jingwang@shsmu.edu.cn

Jingjing Ben, Email: bjj@njmu.edu.cn

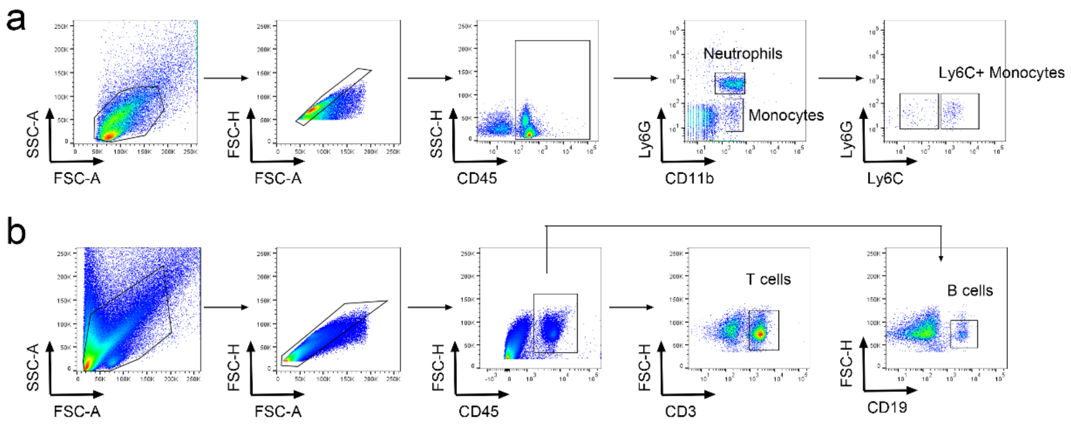

Supplementary Figure 1. Flow cytometry analysis for blood leukocyte populations in mice. (**a**) Gating strategy for neutrophils ($CD45^{+}$ $CD11b^{+}$ $Ly6G^{+}$), monocytes ($CD45^{+}$ $CD11b^{+}$ $Ly6G^{-}$), and $Ly6C^{+}$ monocytes ($CD45^{+}$ $CD11b^{+}$ $Ly6G^{-}$ $Ly6C^{+}$). (**b**) Gating strategy for T cells ($CD45^{+}$ $CD3^{+}$) and B cells ($CD45^{+}$ $CD19^{+}$).

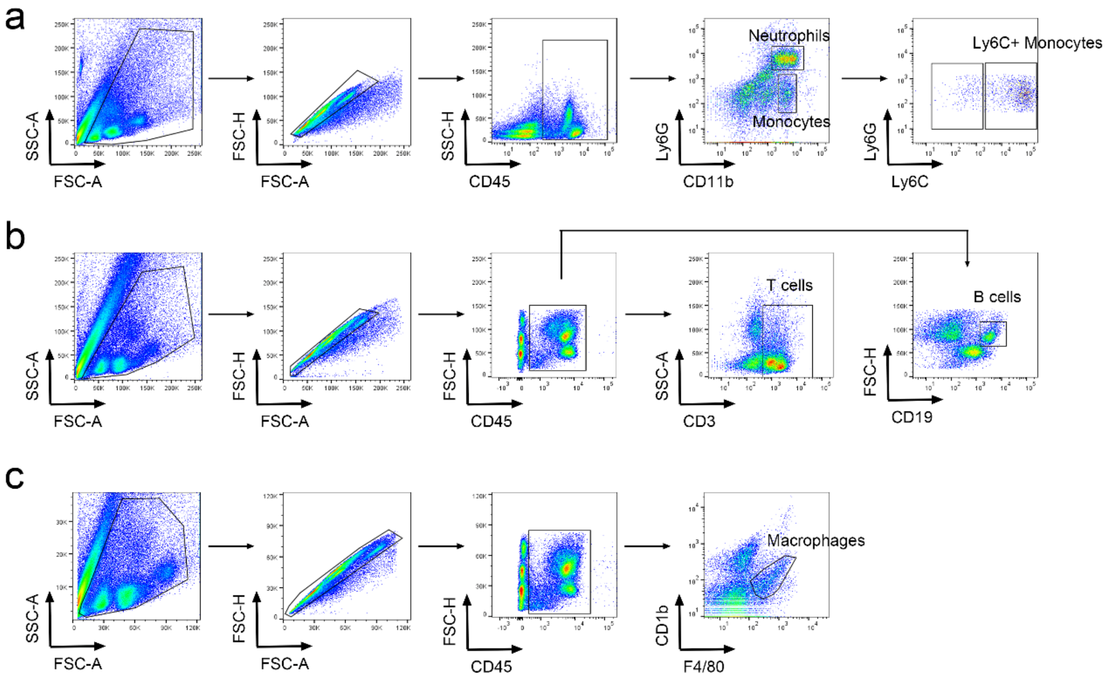

Supplementary Figure 2. Flow cytometry analysis for liver leukocyte populations in mice. (**a**) Gating strategy for neutrophils ($CD45^+$ $CD11b^+$ $Ly6G^+$), monocytes ($CD45^+$ $CD11b^+$ $Ly6G^-$), and $Ly6C^+$ monocytes ($CD45^+$ $CD11b^+$ $Ly6G^-$ $Ly6C^+$). (**b**) Gating strategy for T cells ($CD45^+$ $CD3^+$) and B cells ($CD45^+$ $CD19^+$). (**c**) Gating strategy for macrophages ($CD45^+$ $F4/80^+$ $CD11b^{int}$).

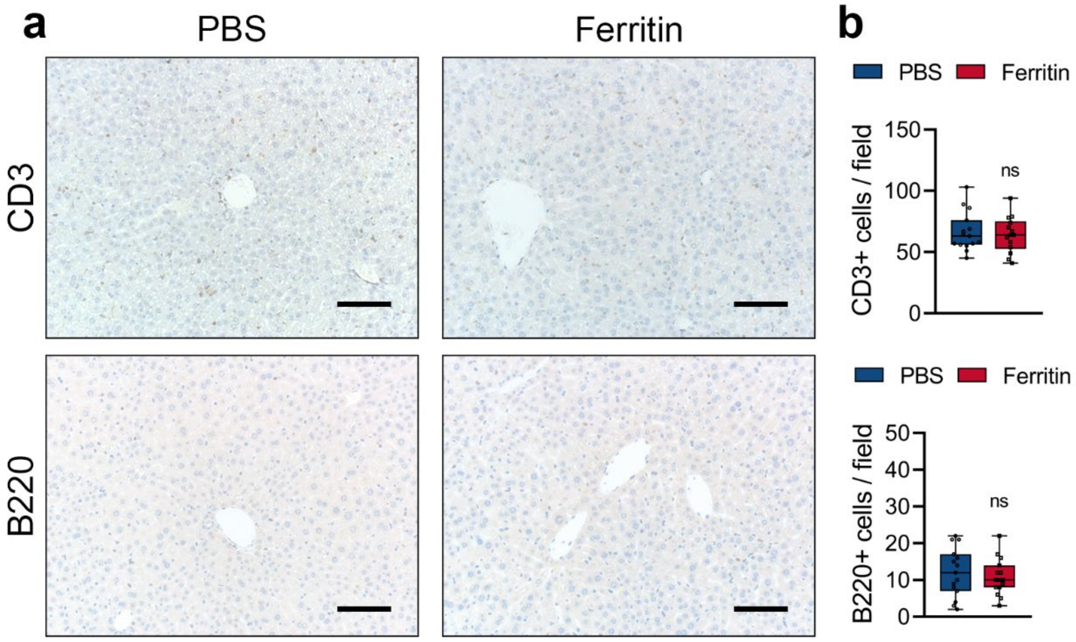

Supplementary Figure 3. Ferritin induces liver injury with neutrophilic inflammation in vivo. (**a**) T cell (CD3) and B cell (B220) infiltrations in the livers from ferritin-treated mice for 6 h were stained by immunohistochemistry. Representative sections are shown. Scale bars, 50 μm. (**b**) The percentage of positive cells was quantified (CD3: n = 15 PBS fields and 14 ferritin fields from 3 independent mice; B220: n = 15 PBS fields and 15 ferritin fields from 3 independent mice). Unpaired two-sided Student's t test; Data are shown as box and whiskers with minima to maxima. ns, not significant.

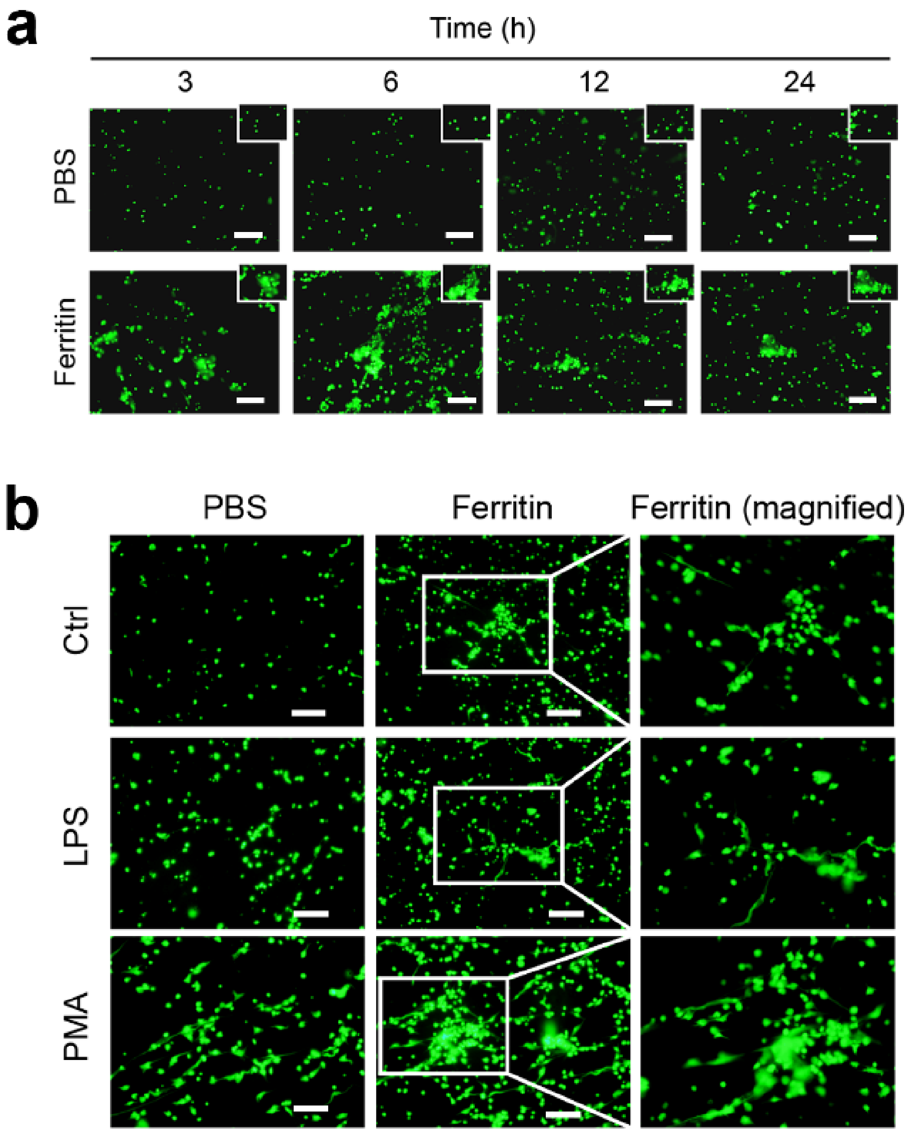

Supplementary Figure 4. Ferritin prompts neutrophils to generate NETs in vivo. (**a**) Representative images of spontaneous NET formation of BMDNs from control mice and ferritin-treated mice at different time points. Scale bars, 100 μm. (**b**) Representative images of spontaneous, LPS- and PMA-induced NET formation of BMDNs from control mice and ferritin-treated mice at 6 h. One representative image of three mice per group was shown. Scale bars, 100 μm.

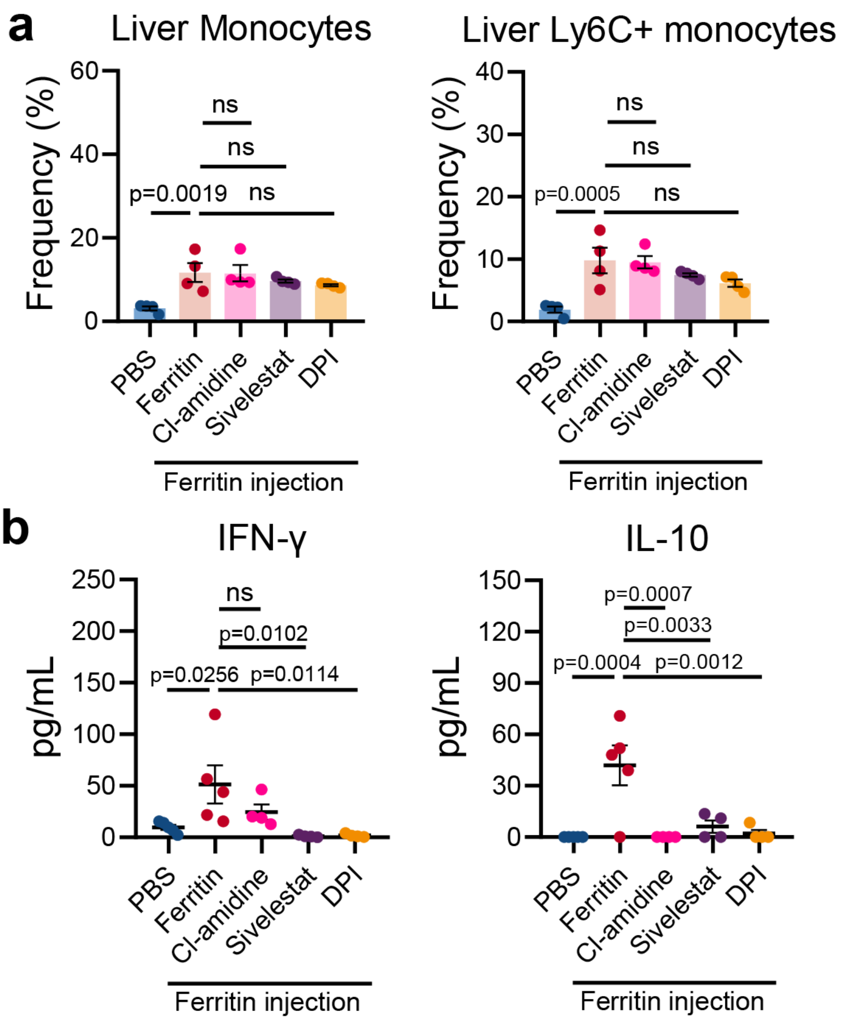

Supplementary Figure 5. Ferritin-induced NETosis and inflammation is dependent on PAD4, NE and ROS. (**a**) Liver monocytes infiltrations were assessed by flow cytometry after treatment with Cl-amidine, sivelestat or DPI in ferritin-treated mice at 6 h (n = 4). (**b**) Serum levels of IFN-γ and IL-10 after treatment with Cl-amidine, sivelestat or DPI in ferritin-treated mice at 6 h (n = 5 in PBS and ferritin groups, n = 4 in other groups). Data are presented as means ± SEM; ns, not significant; one-way ANOVA with Bonferroni’s multiple comparison test.

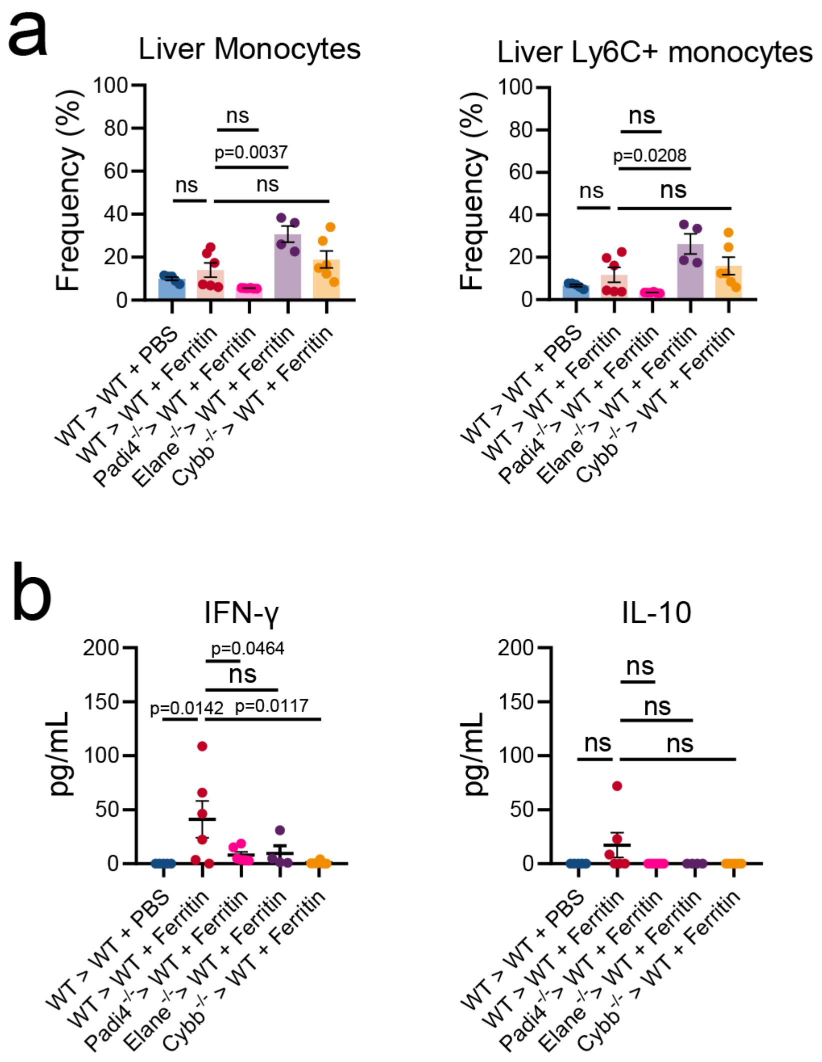

Supplementary Figure 6. Bone marrow transplantations of PAD4$^{-/-}$, NE$^{-/-}$ and ROS$^{-/-}$ mice improves ferritin-induced inflammation. (**a**) Liver monocytes infiltrations were assessed by flow cytometry (WT > WT + PBS: n = 5, WT > WT + Ferritin: n = 6, Padi4-/- > WT + Ferritin: n = 6, Elane-/- > WT + Ferritin: n = 4, Cybb-/- > WT + Ferritin: n = 6). (**b**) Serum levels of IFN-γ and IL-10 (WT > WT + PBS: n = 5, WT > WT + Ferritin: n = 6, Padi4-/- > WT + Ferritin: n = 6, Elane-/- > WT + Ferritin: n = 4, Cybb-/- > WT + Ferritin: n = 6). Data are presented as means ± SEM; ns, not significant; one-way ANOVA with Bonferroni's multiple comparison test.

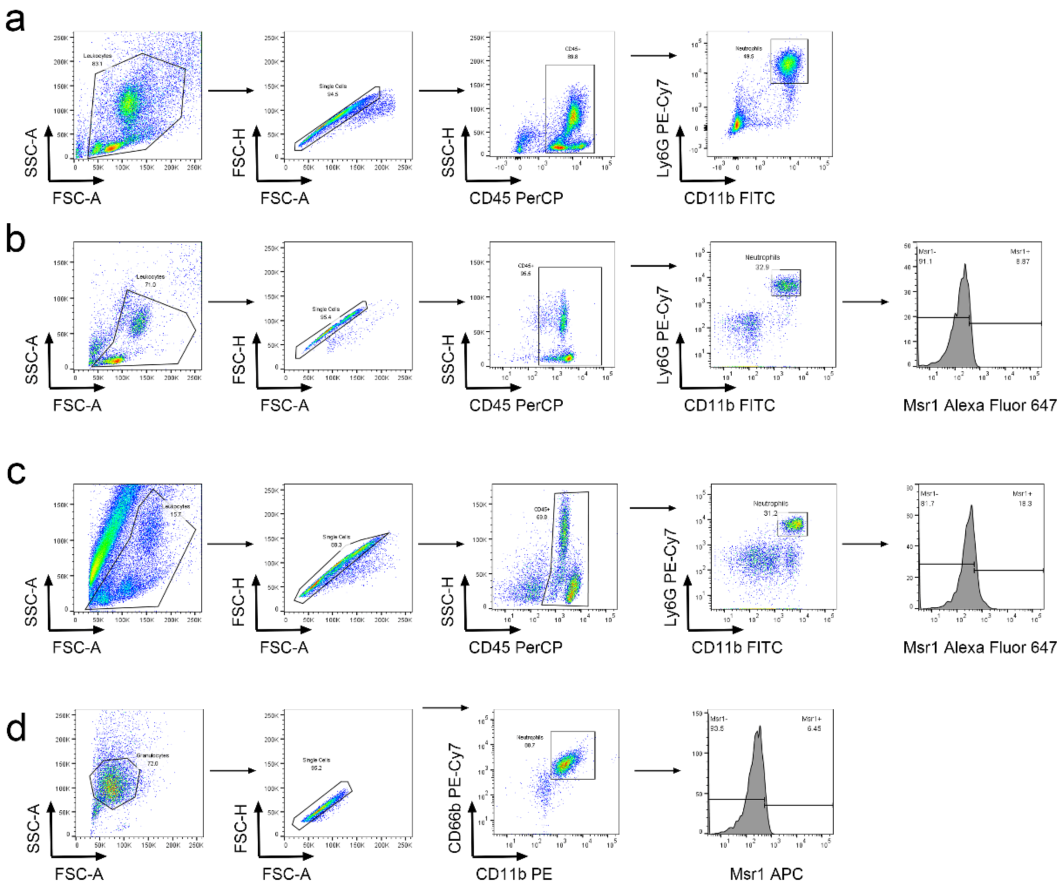

Supplementary Figure 7. Msr1 expression is increased in ferritin-induced inflammation. (**a**) Sorting strategy for bone marrow neutrophils ($CD45^+$ $CD11b^+$ $Ly6G^+$). (**b**) Surface expression of Msr1 on blood neutrophils ($CD45^+$ $CD11b^+$ $Ly6G^+$) was examined. (**c**) Surface expression of Msr1 on liver neutrophils ($CD45^+$ $CD11b^+$ $Ly6G^+$) was examined. (**d**) Surface expression of Msr1 on human neutrophils ($CD11b^+$ $CD66b^+$) after ferritin stimulation.

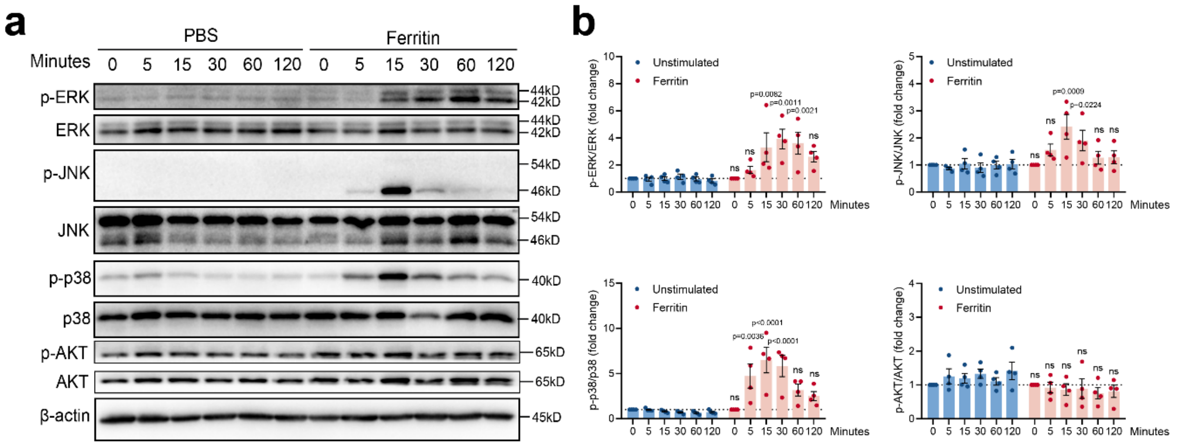

Supplementary Figure 8. MAPK pathway in human neutrophils is activated by ferritin.

(**a**)Western blotting of neutrophils from healthy controls for ERK, JNK, p38 and Akt activation with ferritin stimulation. (**b**) Densitometric analysis of p-ERK/total ERK, p-JNK/total JNK, p-p38/total p38 and p-Akt/total Akt (n = 4 biologically independent experiments). Data are presented as means ± SEM; ns, not significant; two-way ANOVA with Bonferroni's multiple comparison test.

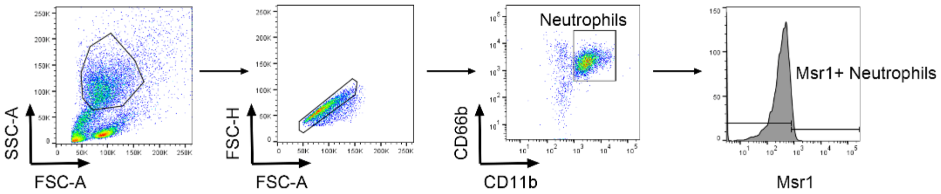

Supplementary Figure 9. Msr1 expression is increased on neutrophils from patients with AOSD. Surface expression of Msr1 on human blood neutrophils (CD11b^{+} CD66b^{+}) was examined.

Supplementary Table 1. Demographic and clinical characteristics of individuals with AOSD.

	**AOSD (n=64)**	
	**Active (n=45)**	**Inactive (n=19)**
Age (Years)	36.0 ± 13.7	38.7 ± 13.3
Sex (F/M)	36/9	16/3
Clinical Manifestations		
Fever	40 (88.9)	0
Arthralgia	38 (84.4)	0
Skin rash	39 (86.7)	0
Sore throat	24 (53.3)	0
Lymphadenopathy	30 (66.7)	0
Splenomegaly	22 (48.9)	0
Hepatomegaly	2 (4.4)	0
Myalgia	12 (26.7)	0
Pericarditis	5 (11.1)	0
Pleuritis	8 (17.8)	0
Laboratory features		
Hemoglobin (g/L)	114.8 ± 17.8	116.2 ± 20.4
Leukocytes (10^9/L)	13.6 ± 6.8	11.5 ± 6.0
Platelets (10^9/L)	243.2 ± 102.4	227.7 ± 88.2
ESR (mm/h)	56.5 ± 40.0	37.2 ± 26.4
CRP (mg/L)	65.4 ± 59.5	37.5 ± 28.4
ALT (U/L)	47.8 ± 48.6	46.2 ± 49.4
AST (U/L)	54.6 ± 49.7	34.3 ± 20.3
Ferritin (>1500 ng/mL)	30 (66.7)	1 (5.3)

Abbreviations: AOSD, adult-onset Still's disease; ESR, erythrocyte sedimentation rate; CRP, C-reactive protein; ALT, alanine transaminase; AST, aspartate transaminase.

* All values are presented as numbers (with percentage) or mean ± SD (standard deviation).

Supplementary Table 2. Primers used in this study.

Gene	Sequence (5’ > 3’)	Species
**β-actin**	Forward: CTACCTCATGAAGATCCTGACC<br>Reverse: CACAGCTTCTCTTTGATGTCAC	Mouse
**Ly6G**	Forward: GACTTCCTGCAACACAACTACC<br>Reverse: ACAGCATTACCAGTGATCTCAGT	Mouse
**IL-1β**	Forward: GCAACTGTTCCTGAACTCAACT<br>Reverse: ATCTTTTGGGGTCCGTCAACT	Mouse
**iNOS**	Forward: GGAGTGACGGCAAACATGACT<br>Reverse: TCGATGCACAACTGGGTGAAC	Mouse
**IFN-γ**	Forward: ATGAACGCTACACACTGCATC<br>Reverse: CCATCCTTTTGCCAGTTCCTC	Mouse
**IL-10**	Forward: CTTACTGACTGGCATGAGGATCA<br>Reverse: GCAGCTCTAGGAGCATGTGG	Mouse
**IL-6**	Forward: CTGCAAGAGACTTCCATCCAG<br>Reverse: AGTGGTATAGACAGGTCTGTTGG	Mouse
**TNF-α**	Forward: CCCTCACACTCAGATCATCTTCT<br>Reverse: GCTACGACGTGGGCTACAG	Mouse
**Emr1**	Forward: TGACTCACCTTGTGGTCCTAA<br>Reverse: CTTCCCAGAATCCAGTCTTTCC	Mouse
**PPAR-γ**	Forward: CTTGGCTGCGCTTACGAAGA<br>Reverse: GAAAGCTCGTCCACGTCAGAC	Mouse
**Arg1**	Forward: CTCCAAGCCAAAGTCCTTAGAG<br>Reverse: AGGAGCTGTCATTAGGGACATC	Mouse
**TGF-β**	Forward: CTTCAATACGTCAGACATTCGGG<br>Reverse: GTAACGCCAGGAATTGTTGCTA	Mouse
**CD163**	Forward: ATGGGTGGACACAGAATGGTT<br>Reverse: CAGGAGCGTTAGTGACAGCAG	Mouse
**CD206**	Forward: CTCTGTTCAGCTATTGGACGC<br>Reverse: CGGAATTTCTGGGATTCAGCTTC	Mouse
**Msr1**	Forward: TGGAGGAGAGAATCGAAAGCA<br>Reverse: CTGGACTGACGAAATCAAGGAA	Mouse
**Scara5**	Forward: TGGGAAGCTAGGGGCTACG<br>Reverse: CGGCAACATTCAGCTCTCTCT	Mouse
**Timd2**	Forward: TGGAATCGTTCCTATGTGTTGG<br>Reverse: TGACCGTATATCCATTGGTCCAG	Mouse
**Tfrc**	Forward: GTTTCTGCCAGCCCCTTATTAT<br>Reverse: GCAAGGAAAGGATATGCAGCA	Mouse
**GAPDH**	Forward: CTGGGCTACACTGAGCACC<br>Reverse: AAGTGGTCGTTGAGGGCAATG	Human
**Msr1**	Forward: TAGGCACTTGGGATGTCTGA<br>Reverse: GTCCTCAATTTGTATTGGTGCT	Human
