# Supplementary material for: Ferritin triggers neutrophil extracellular trap-mediated cytokine storm through Msr1 contributing to adult-onset Still’s disease pathogenesis
Source: Nat Commun. 2022 Nov 10;13:6804. doi: 10.1038/s41467-022-34560-7 (PMC9648446; doi:10.1038/s41467-022-34560-7)
Supplement: Supplementary file 3 — Description of Additional Supplementary Files [file 41467_2022_34560_MOESM3_ESM.pdf]

Description of Additional Supplementary Files

File Name: Supplementary Movie 1
Description: Intravital visualization for neutrophils and extracellular DNA in liver from control mice. PE-conjugated anti-Ly6G antibody (red) was given intravenously to label neutrophils. Sytox green (green) and Alexa Fluor 647-conjugated anti-NE antibody (magenta) was given intravenously to label extracellular traps. The scale bar indicates 50 µm.

File Name: Supplementary Movie 2
Description: Intravital visualization for neutrophils and extracellular DNA in liver from mice at 6 h post ferritin injection. Extracellular traps were labeled with Sytox green (green) and Alexa Fluor 647-conjugated anti-NE antibody (magenta), and neutrophils labeled with PE-conjugated anti-Ly6G antibody (red). The scale bar indicates 50 µm.
