# Supplementary material for: Ferritin triggers neutrophil extracellular trap-mediated cytokine storm through Msr1 contributing to adult-onset Still’s disease pathogenesis
Source: Nat Commun. 2022 Nov 10;13:6804. doi: 10.1038/s41467-022-34560-7 (PMC9648446; doi:10.1038/s41467-022-34560-7)
Supplement: Supplementary file 6 — Reporting Summary [file 41467_2022_34560_MOESM6_ESM.pdf]

nature portfolio

Corresponding author(s): Qiongyi Hu

Last updated by author(s): Oct 13, 2022

Reporting Summary

Nature Portfolio wishes to improve the reproducibility of the work that we publish. This form provides structure for consistency and transparency in reporting. For further information on Nature Portfolio policies, see our Editorial Policies and the Editorial Policy Checklist.

Statistics

For all statistical analyses, confirm that the following items are present in the figure legend, table legend, main text, or Methods section.

n/a	Confirmed	
☐	☒	The exact sample size (*n*) for each experimental group/condition, given as a discrete number and unit of measurement
☐	☒	A statement on whether measurements were taken from distinct samples or whether the same sample was measured repeatedly
☐	☒	The statistical test(s) used AND whether they are one- or two-sided<br>*Only common tests should be described solely by name; describe more complex techniques in the Methods section.*
☒	☐	A description of all covariates tested
☐	☒	A description of any assumptions or corrections, such as tests of normality and adjustment for multiple comparisons
☐	☒	A full description of the statistical parameters including central tendency (e.g. means) or other basic estimates (e.g. regression coefficient) AND variation (e.g. standard deviation) or associated estimates of uncertainty (e.g. confidence intervals)
☐	☒	For null hypothesis testing, the test statistic (e.g. *F*, *t*, *r*) with confidence intervals, effect sizes, degrees of freedom and *P* value noted<br>*Give P values as exact values whenever suitable.*
☒	☐	For Bayesian analysis, information on the choice of priors and Markov chain Monte Carlo settings
☒	☐	For hierarchical and complex designs, identification of the appropriate level for tests and full reporting of outcomes
☒	☐	Estimates of effect sizes (e.g. Cohen's *d*, Pearson's *r*), indicating how they were calculated

Our web collection on statistics for biologists contains articles on many of the points above.

Software and code

Policy information about availability of computer code

Data collection	HISAT2 (v2.0.4) was used to map the clean reads to the genome. Bowtie2 (v2.2.5) was applied to align the clean reads to the reference coding gene set. RSEM (v1.2.12) was used to calculate the expression level of gene. DESeq2(v1.4.5) was used to perform differential expression analysis.
Data analysis	Flow cytometry analysis were performed with FlowJo (version 10.4, Tree Star, Ashland, USA). Immunofluorescence images were acquired using Olympus microscope (IX73) or FV3000 confocal system (Olympus) and analyzed using the ImageJ (version 1.53c, Bio-Rad, USA). Fiji software on Image J (v1.53c) was used to create intravital movies. Western blot data were analyzed using ImageJ (v1.48 & v1.53c). Statistics: GraphPad Prism 8 for PC, SPSS (version 20.0)

For manuscripts utilizing custom algorithms or software that are central to the research but not yet described in published literature, software must be made available to editors and reviewers. We strongly encourage code deposition in a community repository (e.g. GitHub). See the Nature Portfolio guidelines for submitting code & software for further information.

Data

Policy information about availability of data

All manuscripts must include a data availability statement. This statement should provide the following information, where applicable:

- Accession codes, unique identifiers, or web links for publicly available datasets
- A description of any restrictions on data availability
- For clinical datasets or third party data, please ensure that the statement adheres to our policy

The RNA sequencing data generated in this study have been deposited in the GenBank (Gene Expression Omnibus; GEO) under accession code GSE179679 [https://

www.ncbi.nlm.nih.gov/geo/query/acc.cgi?acc=GSE179679]. All the data supporting the findings of this study are available within the article and its supplementary information files, or can be obtained from the corresponding author upon reasonable request. Source data are provided with this paper.

Field-specific reporting

Please select the one below that is the best fit for your research. If you are not sure, read the appropriate sections before making your selection.

☒ Life sciences ☐ Behavioural & social sciences ☐ Ecological, evolutionary & environmental sciences

For a reference copy of the document with all sections, see nature.com/documents/nr-reporting-summary-flat.pdf

Life sciences study design

All studies must disclose on these points even when the disclosure is negative.

Sample size	Sample size was indicated in each figure legend. The sample size was determined based on the level of the heterogeneity of the samples, the expected and observed difference, and our previous researches (PMID: 30616678, 29154963). In the in vivo experiments, 3 to 6 mice/group was sufficient to identify differences between groups with at least 90% power and a 5% significance level.
Data exclusions	No data was excluded from the analyses.
Replication	All experiments were performed independently at least three times using biologically independent replicates except for intravital imaging and human liver biopsies, which were indicated in the figure legends. All replication attempts were successful.
Randomization	A total of 64 AOSD patients (45 active and 19 inactive AOSD patients) admitted to the Department of Rheumatology and Immunology, Ruijin Hospital from May 2017 to December 2018 were consecutively included in the present study. There is no self-selection or group allocation of patient samples for correlation analysis. Female age-matched FVB/n or C57BL/6 mice were randomly allocated into experimental groups.
Blinding	The investigators were blinded to group allocations during data collection and/or analysis. Histological tests were analyzed by an investigator blinded to the experiments.

Reporting for specific materials, systems and methods

We require information from authors about some types of materials, experimental systems and methods used in many studies. Here, indicate whether each material, system or method listed is relevant to your study. If you are not sure if a list item applies to your research, read the appropriate section before selecting a response.

Materials & experimental systems

n/a	Involved in the study	
☐	☒	Antibodies
☒	☐	Eukaryotic cell lines
☒	☐	Palaeontology and archaeology
☐	☒	Animals and other organisms
☐	☒	Human research participants
☒	☐	Clinical data
☒	☐	Dual use research of concern

Methods

n/a	Involved in the study	
☒	☐	ChIP-seq
☐	☒	Flow cytometry
☒	☐	MRI-based neuroimaging

Antibodies

Antibodies used

InVivoMAb Anti-Mouse Ly6G antibody (clone 1A8, #BE0075, BioXcell)
InVivoMAb rat IgG2a isotype control (clone 2A3, #BE0089, BioXcell)
Rabbit polyclonal to Histone H3 (citrulline R2 + R8 + R17) (#ab5103, Abcam)
Rabbit polyclonal to Neutrophil Elastase (#ab68672, Abcam)
Mouse monoclonal to Myeloperoxidase (2C7, #ab25989, Abcam)
Anti-CD204 antibody (EPR7536, #ab151707, Abcam)
Alexa Fluor 647-conjugated anti-mouse CD204 (2F8, #MCA1322A647, Bio-rad)
Anti-Neutrophil Elastase (G-2, #sc-55549, Santa Cruz)
Anti- Human/Mouse Myeloperoxidase Antibody (AF3667, R&D)
Alexa Fluor 647-conjugated anti-Neutrophil Elastase (G-2, #sc-55549 AF647, Santa Cruz)
PE-conjugated anti-mouse Ly6G (clone 1A8, #127608, BD Pharmingen)
PE-Cy7-conjugated anti-mouse Ly6G (clone 1A8, #560601, BD Pharmingen)
FITC-conjugated anti-mouse CD3 (clone 17A2, #561798, BD Pharmingen)
FITC-conjugated anti-mouse CD11b (clone M1/70, #101206, Biolegend)
PE-conjugated anti-mouse F4/80 (clone BM8, #123110, Biolegend)
PE-Cy7-conjugated anti-mouse Ly6G (clone 1A8, #560601, BD Pharmingen)
APC-conjugated anti-mouse Ly6C (clone HK1.4, #128016, Biolegend)

PerCP-conjugated anti-mouse CD45 (clone 30-F11, #557235, BD Pharmingen)
APC-conjugated anti-mouse CD19 (clone 1D3, #550992,BD Pharmingen)
APC-conjugated anti-human CD204 (clone 7C9C20, #371905, Biolegend)
PE-Cy7-conjugated anti-human CD66b (clone G10F5, #305116, Biolegend)
PE-conjugated anti-human CD11b (clone ICRF44, #555388,BD Pharmingen)
Anti -Ly6g Rabbit pAb (#GB11229, Servicebio)
Anti -F4/80 Rabbit pAb (#GB11027, Servicebio)
Anti -CD3 Rabbit pAb (#GB11014, Servicebio)
Anti -B220 Rabbit pAb (#GB11066, Servicebio)
β-actin Mouse mAB (8H10D10, #3700, CST)
Anti -GAPDH Rabbit pAb (AF1186, Beyotime Institute of Biotechnology)
p38 MAPK Rabbit mAb (D13E1, #8690, CST)
Phospho-p38 MAPK Rabbit mAb (D3F9, #4511, CST)
Erk1/2 Rabbit mAb (137F5, #4695, CST)
Phospho-Erk1/2 Rabbit mAb (D13.14.4E, #4370, CST)
JNK2 Rabbit mAb (56G8, #9258, CST)
Phospho-JNK Rabbit mAb (81E11, #4668, CST)
Akt (pan) Rabbit mAb (C67E7, #4691, CST)
Phospho-Akt (Thr308) Rabbit mAb (C31E5E, #2965, CST)
HRP-conjugated anti-rabbit IgG (#7074S, CST)
HRP-conjugated anti-mouse IgG (#L3032, Signalway Antibody)
Alexa Fluor 594-conjugated rabbit anti-mouse IgG, (#33912ES60, YEASEN)
Alexa Fluor 488-conjugated goat Anti-rabbit IgG (#33106ES60, YEASEN)
Alexa Fluor 647-conjugated rabbit anti-goat IgG (#33713ES60, YEASEN)

Validation

Western Blotting:
Erk1/2 Rabbit mAb (1:1000, 137F5 clone, #4695, CST) Species reactivity: human, mouse; Tested applications: WB, IHC, IF, IP, Flow cytometry
Phospho-Erk Rabbit mAb (1:1000, D13.14.4E clone, #4370, CST) Species reactivity: human, mouse; Tested applications: WB, IHC, IF, IP, Flow cytometry
JNK2 Rabbit mAb(1:1000, 56G8 clone, #9258, CST) Species reactivity: human, mouse; Tested applications: WB
Phospho-JNK Rabbit mAb (1:1000, 81E11 clone, #4668, CST) Species reactivity: human, mouse; Tested applications: WB, IHC, IP
p38 MAPK (1:1000, D13E1 clone, #8690, CST) Species reactivity: human, mouse; Tested applications: WB, IHC, IF, Flow cytometry
Phospho-p38 MAPK Rabbit mAb (1:1000, D3F9 clone, #4511, CST) Species reactivity: human, mouse; Tested applications: WB, IHC, IF, IP, Flow cytometry
AKT(pan) Rabbit mAb (1:1000, C67E7 clone, #4691, CST) Species reactivity: human, mouse; Tested applications: WB, IHC, IF, IP, Flow cytometryrabbit
Phospho-AKT (Thr308) Rabbit mAb (1:1000, C31E5E clone, #2965, CST) Species reactivity: human, mouse; Tested applications: WB
Msr1 (CD204) Rabbit mAb (1:1000, #ab151707, Abcam) Species reactivity: human, mouse; Tested applications: WB
Neutrophil Elastase Rabbit pAb (1:1000, #ab68672, Abcam) Species reactivity: human, mouse; Tested applications: WB, IHC
Histone H3 (citrulline R2 + R8 + R17) Rabbit pAb (1:1000, #ab5103, Abcam) Species reactivity: human, mouse; Tested applications: WB, ICC, IP
GAPDH Rabbit mAb (1:1000, AF1186, Beyotime Institute of Biotechnology, Shanghai, China) Species reactivity: human, mouse; Tested applications: WB, IHC, IF, Flow cytometry, IP, ICC
β-actin Mouse mAb (1:1000, #3700S, CST) Species reactivity: human, mouse; Tested applications: WB, IHC, IF, Flow cytometry
HRP-conjugated anti-rabbit IgG (1:5000, #7074S, CST) Species reactivity: rabbit; Tested applications: WB
HRP-conjugated anti-mouse IgG (1:5000, #L3032, Signalway Antibody) Species reactivity: mouse; Tested applications: WB, IHC, ELISA

In vivo treatment:
InVivoMAb Anti-Mouse Ly6G antibody (clone 1A8, #BE0075, BioXcell)
InVivoMAb rat IgG2a isotype control (clone 2A3, #BE0089, BioXcell)

NET quantification:
Neutrophil Elastase Rabbit pAb (1:2000, #ab68672, Abcam) Species reactivity: human, mouse; Tested applications: WB, IHC
Histone H3 (citrulline R2 + R8 + R17) Rabbit pAb (1:1000, #ab5103, Abcam) Species reactivity: human, mouse; Tested applications: WB, ICC, IP
Myeloperoxidase Mouse mAb (1:1000, #ab25989, Abcam) Species reactivity: human; Tested applications: Flow cytometry. This antibody has been validated for IF, ELISA, IHC in both human and mouse in our and other labs. (Pircher, J., Czermak, T., et al., April 2018, Nature Communications; Odqvist, L., Jevnikar, Z., et al., October 2019, Annals of the Rheumatic Diseases)

Immunofluorescence:
Histone H3 (citrulline R2 + R8 + R17) Rabbit pAb (1:200, #ab5103, Abcam) Species reactivity: human, mouse; Tested applications: WB, ICC, IP
Neutrophil Elastase Mouse mAb (1:50, clone G-2, #sc-55549, Santa Cruz) Species reactivity: human; Tested applications: WB, ICC, IP, IF, IHC, ELISA. This antibody has been validated in mouse experiments (Ortiz-Muñoz, G., Yu, M. A., et al., April 2020, The Journal of Clinical Investigation; Ding, Y., Ouyang, Z., et al., March 2022, MedComm)
Myeloperoxidase goat mAb (1:200, #AF3667, R&D) Species reactivity: human, mouse; Tested applications: WB, ICC, IHC
Alexa Fluor 594-conjugated rabbit anti-mouse IgG (1:200, #33912ES60, YEASEN, Shanghai, China) Species reactivity: mouse; Tested applications: ICC, IF, IHC, Flow cytometry
Alexa Fluor 488-conjugated goat Anti-rabbit IgG (1:200, #33106ES60, YEASEN, Shanghai, China) Species reactivity: rabbit; Tested applications: ICC, IF, IHC, Flow cytometry
Alexa Fluor 647-conjugated rabbit anti-goat IgG (1:200, #33713ES60, YEASEN, Shanghai, China) Species reactivity: goat; Tested applications: ICC, IF, IHC, Flow cytometry

IHC:
Anti -Ly6g Rabbit pAb (1:1000, #GB11229, Servicebio) Species reactivity: mouse; Tested applications: IF, IHC

Anti -F4/80 Rabbit pAb (1:1000, #GB11027, Servicebio) Species reactivity: mouse; Tested applications: IF, IHC
Anti -CD3 Rabbit pAb (1:1000, #GB11014, Servicebio) Species reactivity: human, mouse; Tested applications: WB, IF, IHC
Anti -B220 Rabbit pAb (1:4000, #GB11066, Servicebio) Species reactivity: mouse; Tested applications: IF, IHC

Flow cytometry:
PerCP-conjugated anti-mouse CD45 (30-F11 clone, 1:100, #557235, BD) Species reactivity: mouse; Tested applications: Flow cytometry
FITC-conjugated anti-mouse CD11b (M1/70 clone, 1:100, #101206, Biolegend) Species reactivity: human, mouse; Tested applications: Flow cytometry
PE-Cy7-conjugated anti-mouse Ly6G (1A8 clone, 1:100, #560601, BD) Species reactivity: mouse; Tested applications: Flow cytometry
APC-conjugated anti-mouse Ly6C (HK1.4 clone, 1:100, #128016, BD) Species reactivity: mouse; Tested applications: Flow cytometry
FITC-conjugated anti-mouse CD3 (17A2 clone, 1:100, #561798, BD) Species reactivity: mouse; Tested applications: Flow cytometry
PE-Cy7-conjugated anti-mouse CD4 (RM4-5 clone, 1:100, #552775, BD) Species reactivity: mouse; Tested applications: Flow cytometry
APC-H7-conjugated anti-mouse CD8a (53-6.7 clone, 1:100, #560182, BD) Species reactivity: mouse; Tested applications: Flow cytometry
APC-conjugated anti-mouse CD19 (1D3 clone, 1:100, #550992, BD) Species reactivity: mouse; Tested applications: Flow cytometry
PE-conjugated anti-mouse CD49b (DX5 clone, 1:100, #553858, BD) Species reactivity: mouse; Tested applications: Flow cytometry
PE-conjugated anti-mouse F4/80 (BM8 clone, 1:100, 123110, Biolegend) Species reactivity: mouse; Tested applications: Flow cytometry
Alexa Fluor 647-conjugated anti-mouse Msr1 (2F8 clone, 1:100, #MCA1322A647, AbD Serotec, NC, USA) Species reactivity: mouse; Tested applications: WB, ELISA, IHC, IF, IP, Flow cytometry
PE-conjugated anti-human CD11b (ICRF44 clone, 1:100, #555388, BD) Species reactivity: human; Tested applications: Flow cytometry
PE-Cy7-conjugated anti-human CD66b (G10F5 clone, 1:100, #305116, Biolegend) Species reactivity: human; Tested applications: Flow cytometry
APC-conjugated anti-human Msr1 (7C9C20 clone, 1:100, #371905, Biolegend) Species reactivity: human; Tested applications: Flow cytometry

Intravital imaging
PE anti-mouse Ly6G (1A8 clone, #127608, Biolegend) Species reactivity: mouse; Tested applications: Flow cytometry
Neutrophil Elastase Mouse mAb (Alexa Fluor 647, G-2 clone, #sc-55549 AF647, Santa Cruz). Species reactivity: human; Tested applications: WB, ICC, IP, IF, IHC, ELISA. This antibody has been validated in mouse experiments (Ortiz-Muñoz, G., Yu, M. A., et al., April 2020, The Journal of Clinical Investigation; Ding, Y., Ouyang, Z., et al., March 2022, MedComm)

Animals and other organisms

Policy information about studies involving animals; ARRIVE guidelines recommended for reporting animal research

Laboratory animals	Female, 8-12 weeks wild type (WT) FVB/n (#215), and C57BL/6 (#219) mice were purchased from Vital River Laboratories (Beijing, China). Msr1-deficient (Msr1-/-) mice on a C57BL/6 background were provided from Prof. Jingjing Ben in Nanjing Medical University (Nanjing, China), which were purchased from Jackson Laboratory (#006096, RRID: IMSR_JAX: 006096). Padi4-/-, Elane-/- and Cybb-/- mice were obtained from Shanghai Model organisms (Padi4-/-: #NM-KO-190334, Elane-/-: #NM-KO-201544, Cybb-/-: #NM-KO-18031). Animals were maintained under pathogen-free conditions and housed with no more than five animals per cage under a 12-h light/dark cycle with free access to mouse chow and water, ambient temperature 22-24°C and humidity 50-70%. All experiments were performed on sex-and 8 to 12-week-old age-matched animals.
Wild animals	The study did not involve wild animals.
Field-collected samples	The study did not involve samples collected from the field.
Ethics oversight	All experimental protocols described in this study were approved by the Animal Care Committee of Shanghai Jiao Tong University School of Medicine.

Note that full information on the approval of the study protocol must also be provided in the manuscript.

Human research participants

Policy information about studies involving human research participants

Population characteristics	For serum NET detection, Supplementary Table 1 shows the main characteristics of AOSD patients at the time of the blood sampling.<br>For peripheral neutrophil isolation and in vitro experiments, AOSD patients and healthy donors were age and sex-matched (20 to 55-year old, 80% female, 100% Han population).
Recruitment	For serum NET detection, a total of 64 AOSD patients (45 active and 19 inactive AOSD patients) admitted to the Department of Rheumatology and Immunology, Ruijin Hospital from May 2017 to December 2018 were consecutively included in the present study, and serum samples were collected from all participants.<br>For peripheral neutrophil isolation and in vitro experiments, recruitment was performed by the staff of Department of Rheumatology and Immunology, Ruijin Hospital, Shanghai Jiao Tong University School of Medicine. AOSD patients was randomly included for blood collection. The recruitment of healthy donors is not limited to Ruijin Hospital employees, but to others in the community. Individuals volunteers either find the on-line call by themselves or respond to notices which are regularly sent out to recruit donors from the community.

Ethics oversight

Biological samples were obtained under a protocol approved by the Institutional Research Ethics Committee of Ruijin Hospital (ID: 2016–62), Shanghai, China., and all the participants provided informed consent.

Note that full information on the approval of the study protocol must also be provided in the manuscript.

Flow Cytometry

Plots

Confirm that:

☒ The axis labels state the marker and fluorochrome used (e.g. CD4-FITC).

☒ The axis scales are clearly visible. Include numbers along axes only for bottom left plot of group (a 'group' is an analysis of identical markers).

☒ All plots are contour plots with outliers or pseudocolor plots.

☒ A numerical value for number of cells or percentage (with statistics) is provided.

Methodology

Sample preparation

Livers were enzymatically digested using DNase and Collagenase IV for 20 min. Blood was isolated from mice. Intrahepatic and blood leukocytes were stained for 15 minutes at 4°C using fluorescently labeled antibodies: CD45 (30-F11), CD11b (M1/70), Ly6G (1A8), Ly6C (HK1.4), CD3 (17A2), CD4 (RM4-5), CD8a (53-6.7), CD19 (1D3), CD49b (DX5), F4/80 (BM8) (from BD and BioLegend, USA) and Msr1 (AbD Serotec, NC, USA). Human blood were stained for 15 minutes at 4°C using fluorescently labeled antibodies: CD11b (ICRF44), CD66b (G10F5), Msr1 (7C9C20).

Instrument

FACS Canto II cytometer (BD Biosciences); FACSAria for cell sorting

Software

FlowJo software (Tree Star Inc., Ashland, OR)

Cell population abundance

Final sorted cells were >30 % of the single cell population and at least 100000 cells were acquired for qRT-PCR.

Gating strategy

Leukocytes were first identified based on FSC/SSC profiles. Then, doublets were excluded using single-cell gating based on FSC-Height/FSC-Area. Mouse neutrophils were identified as CD45+CD11b+Ly6G+ cells and monocytes were identified as CD45+CD11b+Ly6G- cells and then divided into Ly6C+ monocytes and Ly6C- monocytes. Mouse T cells were identified as CD45+CD3+ cells and B cells as CD45+CD19+ cells. Mouse liver macrophages were selected as CD45+CD11bintF4/80+ cells. Human neutrophils were identified as CD11b+CD66b+ cells.

☒ Tick this box to confirm that a figure exemplifying the gating strategy is provided in the Supplementary Information.
